# Iranian and Iraqi torture survivors in Finland and Sweden: findings from two population-based studies

**DOI:** 10.1093/eurpub/ckab037

**Published:** 2021-04-05

**Authors:** Ferdinand Garoff, Petter Tinghög, Jaana Suvisaari, Eero Lilja, Anu E Castaneda

**Affiliations:** 1 Faculty of Medicine/Psychology, University of Helsinki, Helsinki, Finland; 2 National Institute for Health and Welfare, Helsinki, Finland; 3 Department of Health Sciences, Red Cross University College, Stockholm, Sweden; 4 Division of Insurance Medicine, Department of Clinical Neuroscience, Karolinska Institutet, Stockholm, Sweden

## Abstract

**Background:**

Most refugees and other forced migrants have experienced potentially traumatic events (PTEs). Torture and other traumatic experiences, as well as various daily stressors, impact the mental health and psychosocial well-being of war-affected populations.

**Methods:**

The study includes two population-based samples of Iranian and Iraqi men living in Finland and Sweden. The Finnish Migrant Health and Well-being Study (Maamu) was conducted in 2010–2012. The Linköping study was conducted in Sweden in 2005. In both samples, health and well-being measures, social and economic outcomes as well as health service utilization were reported.

**Results:**

The final sample for analysis consisted of two groups of males of Iranian or Iraqi origin: 278 residents in Finland and 267 residents in Sweden. Both groups were subdivided according to the reported PTEs: Torture survivors; Other PTEs; No PTEs. Migrants that reported PTEs, torture survivors in particular, had significantly poorer social and health outcomes. Torture survivors also reported lower trust and confidence in authorities and public service providers, as well as more loneliness, social isolation and experiences of discrimination.

**Conclusions:**

Torture and other PTEs prevalent in refugee and migrant populations create a wide-ranging and long-term impact in terms of increased risk of various types of adverse social and health conditions. Early identification through systematic and effective screening should be the first step in guiding migrants and refugees suffering from experiences of torture and other PTEs to flexible, multidisciplinary services.

## Introduction

### Torture and other potentially traumatic events

Torture is defined as a political act causing severe pain or suffering, inflicted by or with the acquiescence of a public official, with the intent and purpose of extracting a confession or information, punishment, intimidation, coercion or discrimination.^1^

Most asylum seekers and other forced migrants in high-income countries have experienced at least some type of potentially traumatic event (PTE), either before or during migration,^2–4^ although reported prevalence rates vary between groups of forced migrants^5^ and studies.^6^ During health examinations in Tampere, Finland, 57% of 170 adult asylum seekers from 25 different countries reported torture experiences and 12% reported other experiences of violence.^7^ The prevalence rates of torture among adult forced migrants living in high-income countries varies greatly between studies, but is generally higher in men and in older age groups. Torture often occurs in a context of several other war-related PTEs.^2^

### Health and social impact of torture

Prevalence rate of post-traumatic stress disorder (PTSD) among torture survivors is higher than for survivors of other types of war trauma.^8^^,^^9^ More than 90% of torture survivors referred to one of 23 torture rehabilitation centres in the US were diagnosed with PTSD or major depressive disorder, and the rates were significantly related to the number and types of torture, asylum seeker immigration status and reporting rape experiences.^3^ In addition to PTSD and major depressive disorder, psychological and cognitive effects of torture can include personality change, confusion, disorientation and impaired memory, along with symptoms such as insomnia or sexual dysfunction.^1^^,^^10^ Torture also frequently leads to chronic pain. Pain is associated with greater severity of both PTSD symptoms and major depression, and intrusive memories and flashbacks can exacerbate existing pain.^11^

Even after many years, past torture is significantly associated with emotional distress.^12^ Guilt and shame about humiliation during torture, as well as guilt of surviving, are common problems that discourage disclosure in health care services.^13^ Health service usage is also impacted by migrant-related factors such as health beliefs and help-seeking behaviour, linguistic barriers, perception and knowledge of the health system, stigma and social anxieties, as well as other social and cultural factors. In addition, health service usage is impacted by service-related factors such as lack of appropriate services and financial or logistical barriers, as well as political, economic and administrative constraints on access to the health services.^14^

### Daily stressors and resource loss

In addition to torture and direct exposure to war-related violence and loss, various daily stressors, as well as chronic fear and vulnerability, impact mental health and psychosocial well-being of war-affected populations.^15^^,^^16^ Chronic daily stress may gradually diminish people’s capacity to cope effectively with potentially traumatic life events.^17^

The conservation of resources theory^18^ conceptualizes this process in terms of resource loss and gain. PTEs may lead to sequela such as loss of health, well-being or other resources. Individuals with less access to resources are more prone to further resource loss under stressful conditions, whereas having more resources can buffer against future resource loss and also facilitate future resource gain.^19^ According to Hollifield et al.,^20^ loss of resources is frequent in the aftermath of trauma and may exacerbate PTSD symptoms in a bidirectional relationship that continues over time.

Evidence of long-term resource loss was found among settled refugees in Norway^21^ and Sweden.^22^ Psychiatric symptoms and suffering was chronic, and the situation was exacerbated by lack of resources post-migration, such as unemployment, housing problems and lack of social contacts, which in turn predicted the maintenance of PTSD symptoms.^21^ The political situation in the home country as well as the well-being of significant others also impacted self-reported health significantly. Participants with PTSD were found to be more affected by negative life events.^22^

### The aim of the study

In this study, we aim to compare the health and well-being, the social and economic situation, and health service utilization of torture survivors, survivors of other trauma and non-traumatized migrants.

We hypothesize that torture and other traumatic experiences put the individual at significant risk to develop a wide range of adverse social and health conditions, including mental health symptoms, that may need to be addressed. PTEs also impact the capacity of migrants to develop new resources, such as language and vocational skills or social networks. We test this hypothesis by comparing the prevalence of a wide-range social and health outcomes among torture survivors, survivors of other trauma and non-traumatized migrants.

## Methods

The findings reported in this article are drawn from two population-based studies conducted in neighbouring Nordic countries with similar, though not identical populations and methodologies; therefore, it was decided to report the findings together in one paper, but to conduct the analyses in parallel. Other findings from these studies have been reported elsewhere.^23–25^

The prevalence of PTEs was low in parts of the study populations, and torture experiences were uncommon among women; therefore, only men of Iranian or Iraqi origin were included in the final study samples used in this article.

### Datasets

In Finland, a comprehensive cross-sectional interview and health examination survey, the Finnish Migrant Health and Well-being Study (Maamu), was conducted in 2010–2012 by the National Institute for Health and Welfare. Stratified sampling was conducted by locality and migrant group by simple random sampling from the National Population Registry among those fulfilling the inclusion criteria. General inclusion criteria were aged between 18 and 64 years and residence in one of six Finnish cities. Participants also had to have resided in Finland for at least a year and not be living in an asylum reception centre. Inclusion criteria for participants relevant for this paper were male gender, Iraq or Iran as country of birth and Kurdish as mother tongue. For the six cities in Finland, the total number of Kurds matching these criteria in 2010 was 2568 (57% men; sampling probability 39%, with slight variation between the cities). In the sample, 574 Kurdish men were included in the sample.

Individual face-to-face interviews on health and well-being and health examinations for this group were conducted by trained study personnel of Kurdish origin who spoke both the language of the target group and Finnish. All male respondents of Kurdish origin that completed the full interview, that included questions about PTEs, were included in the final sample used for this paper (*n* = 278, response rate 48.4%).

In Sweden, a survey was carried out in 2005 in the municipality of Linköping. The survey covered demography, social support, traumatic life events, acculturation strategies and use of healthcare, and several instruments intended to detect poor health with the emphasis on mental health. To be eligible for participation, the respondent had to be between 20 and 75 years old and resident in Sweden for at least three years. Additional inclusion criteria for participants relevant for this paper were male gender and Iraq or Iran as country of birth. However, ethnic origin was not systematically reported; therefore, the proportion of persons of Kurdish background is not known. The government population registry included 1157 eligible males from Iran (573) and Iraq (584). A stratified random sample was drawn that included 274 Iranian (sampling probability 47.8%) and 322 Iraqi (sampling probability 55.1%) men.

The survey questionnaire was sent by mail, and respondents were given the opportunity to answer Arabic or a Farsi version of the questionnaire. All male respondents from Iran (136; response rate 49.6%) and Iraq (131; response rate 40.7%) that completed the full survey, including questions about PTEs, were included in the final sample used for this work (*n* = 267, overall response rate 44.8%).

### Ethics approval and consent to participate

The Finnish study was approved by the Coordinating Ethical Committee of the Helsinki and Uusimaa Hospital District, Finland. The Swedish study was part of a project reviewed and approved by the regional ethics committee in Linköping (Dnr. 191-05), Sweden. Participation was voluntary and approved with informed consent in both studies.

### Measures

#### Potentially traumatic events

Participants were classified into three groups according to self-reported potentially traumatic experiences (PTEs): (1) torture survivors, (2) survivors of other PTEs without experiences of torture and (3) those reporting no PTEs. The PTEs asked about in the Finnish study were experiences of war, natural disaster, seeing violence, sexual violence, physical harm, imprisonment, torture and other severe violence, each answered with ‘yes’ or ‘no’. In the Swedish study, questions were largely the same, but arrest was asked separately, and natural disasters or other severe violence were not asked about. It must be noted that these PTEs are closely interrelated and should not be considered as discrete events,^15^ e.g. in this study, torture frequently occurs during imprisonment or arrest and involves physical harm.

#### Measures of health and well-being, social and economic situation and health service utilization

All outcome measures were classified into binary variables indicating poor outcomes. The variables and classification are summarized in table 1.

**Table 1:** Indicators of poor outcomes

Topic	Description	Classified as poor outcomes if…
Not working		
Finland	Main daily activity (seven options).	Not having a part-time or full-time job.
Sweden	Main daily activity (nine options).	Not having a part-time or full-time job or being self-employed.
Low income		
Finland	Household income per month in euros (nine levels).	850 euros or less per month.
Sweden	Household income per year in SEK.	Less than 100 000 SEK per year.
Poor language skills		
Finland	Comprehension, speaking, reading and writing of Finnish or Swedish rated ‘good’ to ‘not at all’, four-point Likert scale.	At least one of comprehension, speaking, reading or writing in Finnish or Swedish rated as poor or not at all.
Sweden	Use of interpreters in interactions with authorities and health services (yes/no).	Reported use of interpreters in interactions with authorities and health services.
Mental health symptoms		
Finland	HSCL-25,^24^ 25 questions about depression and anxiety symptoms, four-point Likert scale.	Cut-off point ≥ 1.75.^25^
Sweden	HSCL-25,^24^ 25 questions about depression and anxiety symptoms, four-point Likert scale.	Cut-off point ≥ 1.75.^25^
PTSD symptoms		
Sweden	SIP-22,^26^ 22 items, reflecting DSM-III-R criteria for PTSD, four-point Likert scale.	‘Always’ or ‘extremely’ on at least one intrusion, three avoidance and two hyper-arousal questions.^26^
Poor quality of life		
Finland	Quality of life ‘very good’ to ‘very poor’, five-point Likert scale.	Subjective well-being was rated as poor or very poor.
Sweden	WHO-10;^27^ 10 questions to assess the respondents’ subjective well-being, four-point Likert scale.	Cut-off point ≥ 30,^28^
Loneliness/isolation		
Finland	Feeling lonely, ‘never’ to ‘all the time’, five-point Likert scale.	‘Frequently’ or ‘always’ feeling lonely.
Sweden	‘Do you interact with relatives in the city where you live?’ (yes/no) and ‘Do you interact with friends?’ (yes/no)	Neither interacting with friends nor with relatives locally.
Chronic illness		
Finland	Long-term illness or injury that decreases capacity to work or function (yes/no).	Chronic illness or injury reported.
Sweden	Chronic disease (yes/no).	Chronic disease reported.
Pain		
Finland	Pain in the past 7 days (yes/no).	Pain reported.
Injury from violence		
Finland	Permanent injury due to violence (yes/no).	Reported permanent injury due to violence.
Poor health		
Finland	Current state of health rated ‘good’ to ‘poor’, five-point Likert scale.	State of health rated as ‘poor’ or ‘quite poor’.
Sweden	State of health for the past 3 months, rated ‘very good’ to ‘poor’ five-point Likert scale.	State of health rated as ‘poor’ or ‘very poor’.
Smoking daily		
Finland	Current level of smoking, ‘not at all’ to ‘daily’ (three levels).	Currently daily smoking.
Alcohol use		
Finland	Frequency of alcohol use, ‘never’ to ‘4 times per week or more’.	Alcohol use at least two to four times per month.
Visit doctor		
Finland	Visit to a doctor in the past year (yes/no).	Reported visiting a doctor in the past year.
Sweden	Visit to a doctor during the past 6 months (yes/no).	Reported visiting a doctor in the past 6 months.
Mental health service use		
Finland	Use of health services due to mental health problems in the past year (yes/no).	Reported using health services due to mental health problems in the past year.
Sweden	Visiting a psychiatrist in the past 6 months (yes/no).	Reported visiting a psychiatrist in the past 6 months.
Mental health service need		
Finland	Current need of health services due to mental health problems (yes/no).	Reported need of health services due to mental health problems.
Medication (any prescription)		
Finland	Use of any prescription medication in the past year (yes/no).	Reported use of prescription medication in the past year.
Sweden	Use of any prescription medication in the past 6 months (yes/no).	Reported use of prescription medication in the past 6 months.
Medication (painkillers)		
Finland	Prescription for painkillers (opiates, analgesics or anti-inflammatory medication).	Prescription for painkillers.
Sweden	Use of prescription painkillers in the past 6 months (yes/no).	Reported use of prescription painkillers in the past 6 months.
Medication (sleep)		
Finland	Prescription for sleep medication.	Prescription for sleep medication.
Sweden	Use of prescription sleep medication in the past 6 months (yes/no).	Reported use of prescription sleep medication in the past 6 months.
Medication (other mental health)		
Finland	Prescription for psychotropic drugs (antipsychotics, anxiolytics, antidepressants or combination drugs).	Prescription for psychotropic drugs.
Sweden	Use of prescription depression (yes/no) or other mental health medication (yes/no) in the past 6 months.	Reported use of either depression or other mental health medication in the past 6 months.
Trust		
Finland	Trust in authorities (health services, police, social services, employment office, justice system), ‘not at all’ to ‘completely’, four-point Likert scale.	Reported level of trust ‘a little’ or ‘not at all’, separately for the five listed authorities.
Discrimination		
Finland	Experiences of discrimination or unfair treatment (by police, social services, in the street; yes/no/NA).	Reported discrimination or unfair treatment, separately for the three listed places.
Discrimination (daily life)		
Finland	Any experiences of discrimination or unfair treatment by authorities or elsewhere (yes/no/NA).	Any reported experiences of discrimination or unfair treatment.
Sweden	Frequency of discrimination experiences due to ethnic background, ‘never’ to ‘always’, four-point Likert scale.	Reported ‘frequent’ or ‘always’ experiencing discrimination due to ethnic background.

#### Demographic variables

Demographic variables used in the analyses include age, marital status, education level, time in the host country, country of origin, refugee status (in Finland) and age at immigration. These were classified into binary or ternary variables for purposes of analysis. Refugee status was classified if residency had been obtained through granting of refugee status or asylum application.

### Statistical analysis

All analyses were conducted separately for the Finnish and Swedish datasets using SUDAAN 11.0.1 and SAS 9.3 software packages.^26^ Inverse probability weights, based on age group, marital status, country of origin and study location, were applied in all analyses in the Finnish dataset to reduce the effect of non-response and different sampling probabilities.^27^ Similar analysis weights were constructed for the Swedish data based on age group, marital status and country of birth. The stratification of both study samples was accounted for in all analyses. In addition, finite population correction was used because a relatively large portion of the study populations were included in the samples.^28^

Age-adjusted prevalences and confidence intervals of the outcome measures were calculated with logistic regression using predictive margins.^29^ Helmert regression coding was used to compare the differences between the levels of resource variables. Statistical significance of the difference was assessed with Satterthwaite F-statistic.

## Results

### Demographics

Following the process described in the ‘Methods’ section, the final sample for analysis consisted of two groups of males of Iranian or Iraqi origin: 278 residents in Finland and 267 residents in Sweden. Both were subdivided into three groups reporting: No PTEs; Torture; Other PTEs. As has been reported previously,^25^ 34.7% of the Kurdish men in the Finnish sample reported torture experiences (Iranian Kurdish men 36.4%; Iraqi Kurdish men 31.8%). In the Swedish sample, the figure was slightly lower at 26.9% (Iranian men 25.5%; Iraqi men 27.5%). Torture was more prevalent in the older age groups, likely due to migration history and torture practices in Iran and Iraq. Very few participants that had arrived as minors in Finland or Sweden reported torture experiences. Table 2 summarizes the demographics of the two datasets.

**Table 2:** Demographic and migration-related factors of the study populations according to type of PTEs

	Finland				Sweden			
	All (*N* = 278)	No PTE (*N* = 49)	Other PTE (*N* = 136)	Torture (*N* = 93)	All (*N* = 267)	No PTE (*N* = 42)	Other PTE (*N* = 156)	Torture (*N* = 69)
Age								
18–29 (20–29 Sweden)	37.9	64.6	41.5	16.5	15.5	29.9	31.4	8.1
30–44	41.3	33.2	41.3	46.2	59.9	41.1	36.7	35.7
45–64 (45–75 Sweden)	20.8	2.2	17.2	37.2	24.6	29.0	31.9	56.3
Marital status								
Married/living together	63.4	47.3	64.1	72.0	64.7	64.9	65.7	62.2
Others	36.6	52.7	35.9	28.0	35.3	35.1	34.3	37.8
Education								
Started second school	42.3	53.1	41.0	37.8	80.6	84.5	83.2	71.8
Others	57.7	46.9	59.0	62.2	19.4	15.5	16.8	28.2
Time in country								
5 years or less	22.7	21.6	29.6	13.0	11.9	10.0	13.3	9.8
6–14 years	52.1	37.4	48.8	65.9	41.5	25.6	41.6	51.3
15 years or more	25.2	41.0	21.6	21.1	46.6	64.4	45.1	38.9
Country of origin								
Iran	37.1	44.9	32.4	39.5	51.0	41.6	50.4	50.8
Iraq	62.9	55.1	67.6	60.5	49.0	58.4	49.6	49.2
Refugee status								
Refugee/asylum	83.9	72.9	81.9	93.5	–	–	–	–
Other	16.1	27.1	18.1	6.5	–	–	–	–
Age of immigration								
18 years or less	22.3	48.2	23.4	5.3	21.5	28.5	27.3	3.1
Over 18 years	77.7	51.8	76.6	94.7	78.5	71.5	72.7	96.9

### Outcomes

On many of the measures, a common pattern was evident in comparing the three groups: those reporting torture were doing the worst while those reporting other PTEs were doing worse than those reporting no PTEs (table 3). However, even so, this was a general trend rather than a clear-cut differentiation of groups, and on some indicators, there were no group differences. This included employment status, chronic illness or pain and alcohol use. It must be noted, however, that the majority of participants in both studies were not employed, despite all being of working age in the Finnish sample and almost all in the Swedish sample. Most participants had some language skills in Finnish or Swedish, but there was a general trend towards poorer language skills among those reporting torture or other PTEs. Torture in particular, but also other reported PTEs, were also linked with a significant increase in severe anxiety and depression symptoms in both samples and clinical levels of PTSD in Sweden. Torture and other reported PTEs were also linked with poor quality of life as well as increased loneliness in the Finnish sample and poorer social connections in the Swedish sample. In the Swedish sample, chronic illness was more frequently reported by those who reported torture and other PTEs; meanwhile, this was not the case in the Finnish study. Prevalence of chronic pain was not significantly different in the different groups in Finland, but injury from violence was more frequent among torture survivors. Self-ratings of poor health were also more frequent among those reporting torture in particular, but also for other reported PTEs, in both studies. Smoking was more common among those who reported torture and other PTEs in the Finnish sample. In the Finnish sample, only 15% of torture survivors reported mental health service use, whereas 25% expressed a need for mental health services. In the Swedish sample, very few reported having seen a psychiatrist, but visits to the doctor were more frequent among those who reported PTEs. Use of prescription medication was increased among torture survivors, but also to a lesser extent among those reporting other PTEs, in the Swedish sample. Painkiller and sleep medication use was more frequent among those reporting PTEs and torture in particular in the Swedish sample; this was not the case in the Finnish sample where very few reported use of sleep medication. Reported use of other mental health medication was very infrequent in both studies.

**Table 3:** Indicators of poor outcomes by type of PTEs

Resources	No PTE, % (Cl)	Other PTE, % (Cl)	Torture, % (Cl)	No vs. any PTE, *P*-value	Other PTE vs. Torture, *P*-value
Not working					
Finland	51.0 (37.5–64.3)	52.3 (44.5–60.1)	55.8 (45.5–65.6)	0.693	0.600
Sweden	54.1 (40.0–67.6)	63.8 (56.5–70.5)	56.6 (44.7–67.8)	0.436	0.299
Low income					
Finland	26.2 (16.1–39.7)	31.3 (24.7–38.8)	47.7 (37.8–57.8)	0.090	0.012
Sweden	25.6 (14.3–41.7)	45.5 (37.5–53.7)	49.0 (36.1–62.0)	0.017	0.657
Poor language skills					
Finland	7.1 (2.7–17.4)	20.3 (14.7–27.4)	27.0 (18.6–37.4)	0.011	0.249
Sweden	11.2 (4.9–31.2)	17.8 (13.0–23.9)	27.7 (19.5–37.7)	0.065	0.044
Mental health symptoms					
Finland	11.7 (5.0–24.9)	21.5 (15.4–29.3)	36.1 (26.0–47.7)	0.035	0.028
Sweden	17.2 (8.7–23.4)	45.6 (38.3–53.1)	62.3 (50.1–73.0)	<0.001	0.021
PTSD symptoms					
Sweden	4.1 (1.1–14.4)	16.5 (11.6–22.9)	35.8 (25.1–48.0)	0.005	0.002
Poor quality of life					
Finland	10.4 (4.4–22.4)	21.2 (15.4–28.4)	40.7 (30.8–51.5)	0.097	0.010
Sweden	6.0 (1.6–19.7)	20.8 (15.1–27.8)	36.3 (24.7–49.7)	0.011	0.027
Loneliness/isolation					
Finland	8.6 (3.6–19.1)	17.8 (12.6–24.6)	36.1 (26.5–47.0)	0.011	0.003
Sweden	2.4 (NA)	11.4 (7.3–17.3)	25.2 (16.6–36.3)	NA	0.041
Chronic illness					
Finland	25.3 (14.7–39.9)	27.5 (21.0–35.2)	31.8 (23.6–41.4)	0.567	0.456
Sweden	10.1 (4.3–21.9)	24.7 (19.1–31.4)	43.9 (33.9–54.5)	0.001	0.001
Pain					
Finland	47.5 (33.0–62.4)	53.1 (44.9–61.2)	53.6 (43.3–63.7)	0.499	0.942
Injury from violence					
Finland	8.3 (3.4–19.1)	7.4 (4.5–12.1)	29.0 (20.2–39.6)	0.195	<0.001
Poor health					
Finland	4.2 (1.1–14.9)	12.5 (8.1–19.0)	27.9 (19.3–38.6)	0.023	0.005
Sweden	12.7 (5.6–26.3)	23.0 (17.3–29.9)	44.4 (32.8–56.6)	0.012	0.001
Smoking					
Finland	22.8 (13.6–35.6)	29.1 (22.6–35.6)	43.3 (33.4–53.8)	0.026	<0.001
Alcohol use					
Finland	24.0 (14.4–37.3)	27.2 (20.9–34.7)	33.8 (24.5–44.5)	0.376	0.290
Visit doctor					
Finland	71.0 (57.9–81.4)	71.2 (63.6–77.8)	68.7 (58.0–77.6)	0.881	0.680
Sweden	42.6 (28.9–57.5)	60.5 (52.9–67.6)	64.8 (52.6–75.3)	0.017	0.536
Mental health service use					
Finland	4.9 (1.3–17.1)	4.2 (1.8–9.3)	15.0 (7.8–27.1)	0.500	0.020
Sweden	0 (NA)	1.6 (NA)	7.0 (2.9–15.9)	NA	NA
Mental health service need					
Finland	1.9 (0.3–10.0)	6.2 (3.3–11.3)	25.0 (16.3–36.3)	0.026	<0.001
Medication (any prescription)					
Finland	46.0 (33.1–59.5)	67.9 (60.3–74.8)	68.2 (57.3–77.4)	0.004	0.964
Sweden	35.2 (22.7–50.2)	50.2 (42.8–57.7)	65.2 (52.4–76.1)	0.008	0.039
Medication (painkillers)					
Finland	14.7 (8.7–23.9)	19.4 (15.7–23.8)	15.9 (10.8–22.9)	0.542	0.356
Sweden	11.1 (4.8–23.5)	23.1 (17.3–30.0)	32.5 (22.5–44.4)	0.023	0.130
Medication (sleep)					
Finland	2.1 (NA)	1.2 (NA)	3.7 (1.3–10.2)	NA	NA
Sweden	0 (NA)	5.8 (3.1–10.5)	22.4 (14.5–33.0)	NA	0.003
Medication (other mental health)					
Finland	2.6 (NA)	1.9 (0.7–5.3)	6.2 (2.8–13.2)	NA	0.524
Sweden	0 (NA)	2.0 (0.8–5.6)	6.9 (3.0–15.0)	NA	0.179
Trust (in Finland)					
Public health services	15.6 (8.2–27.4)	19.8 (14.2–26.8)	34.8 (25.5–45.4)	0.097	0.010
Police	26.1 (15.7–40.3)	20.2 (14.6–27.4)	42.2 (32.1–53.1)	0.598	0.001
Social services	19.5 (11.1–32.0)	22.9 (16.9–30.2)	44.9 (34.7–55.4)	0.061	0.001
Employment office	27.0 (16.7–40.6)	33.4 (26.3–41.3)	37.8 (28.3–48.4)	0.257	0.496
Justice system	27.9 (16.9–42.5)	16.0 (10.9–22.8)	37.6 (27.4–49.1)	0.726	0.001
Discrimination (in Finland)					
Police	20.2 (10.6–35.0)	21.7 (15.2–30.0)	49.3 (38.0–60.6)	0.088	<0.001
Social services	9.8 (4.2–21.4)	17.3 (12.2–24.0)	33.7 (24.4–44.6)	0.031	0.005
On the street	19.7 (11.2–32.4)	29.8 (23.3–37.3)	36.8 (28.9–49.4)	0.052	0.164
Discrimination (daily life)					
Finland	38.6 (24.4–52.4)	41.6 (34.2–49.4)	61.2 (51.4–70.1)	0.111	0.002
Sweden	22.7 (12.6–37.4)	16.1 (11.3–22.4)	25.6 (22.5–44.4)	0.741	0.085

In the Finnish sample, trust and confidence in authorities and public service providers was significantly lower among torture survivors than in the other groups, and they also reported more discrimination by authorities and experiences of discrimination in daily life. In the Swedish study, one question asked about discrimination due to ethnic background, but it did not yield a significant trend (i.e. *P* < 0.05).

## Discussion

Torture has been linked with reduced resilience, mental health symptoms and poorer quality of life in the long term.^1^^,^^3^^,^^9^^,^^10^^,^^22^^,^^30^ This study shows that torture and other PTEs have long-term consequences on a very wide range of health and social indicators not only mental health symptoms. Participants in this study that reported torture and other PTEs had significantly worse outcomes compared with those not reporting any PTEs. Employment status was not impacted by past PTEs or torture, though it must be noted that the majority in both samples was not employed. The impacts of PTEs are significant and varied, and torture survivors are at particular risk of ending up in situations where resilience is reduced and resource losses accumulate.^20^

These risk-factors are not always recognized in health care services,^31^ and this may be due to hesitation to disclose past PTEs as a result of feelings of guilt and shame linked with the torture events^13^ and with avoidance behaviours linked with post-traumatic stress symptoms. In this study, health service utilization was elevated among torture survivors, but levels were nonetheless quite low, particularly with regard to mental health services. Barriers to accessing health services may also include loss of interpersonal trust^32^ that follows the intentional harm caused in torture. In this study, confidence and trust in authorities and public service providers was significantly lower among torture survivors; they also were more lonely and isolated. Torture survivors also reported more discrimination by authorities and in daily life in the Finnish sample; this may be an indication that they have become sensitized to felt injustices. This can in turn have severe social consequences if it results in a pattern of explosive outbursts or avoidance^33^ or decreased help seeking.

### Limitations

Experiences of torture and other PTEs were based on self-report questions in these studies, with no additional verification. However, self-defined torture has been found to have high sensitivity and specificity when compared with the UN definition of torture among Middle Eastern refugees.^34^ Torture is difficult to define and operationalize,^35^ and severity was not assessed in these studies. Torture is also frequently linked with other PTEs and practiced in situations with other environmental stressors,^36^ making it difficult to assess the impact of torture experiences apart from other stressors in the former home or host country. However, we believe that reports of torture can still function as an important indication of potentially diminished resilience and increased risk of various types of adverse social and health conditions.

The non-response rate was high in both studies, and as noted by Tinghög et al.,^37^ mental health problems are more common among the non-respondents, potentially underestimating symptom levels. However, register-based information was available for all eligible study participants. This allowed for attrition analysis to be conducted. This showed that the sample’s demographic profile fairly closely resembled that of the non-respondents with some exceptions. In addition, non-response weights were constructed to obtain more reliable estimates.

Only age was adjusted for in the logistic regression analyses. Other demographic variables were considered to be included in the analyses; however, differences on some of the variables were small. Also, including variables related to time in the host country or age of migration could make the interpretation of results difficult as PTEs are much less likely to occur in the host country. Therefore, it was decided to only adjust for age in the analyses.

In order to investigate the impact of torture, the study population was limited to men of Iranian or Iraqi origin residing in two Nordic countries, and this limits the generalizability of findings. In addition, ethnic origin was not systematically reported in the Swedish study, making a direct comparison of the two samples difficult. However, the parallel analyses of the two separate samples showed similar patterns in both of the host countries, indicating a degree of stability of the pattern identified.

## Conclusions

The findings of this study corroborate previous findings that torture and other PTEs create a wide-ranging and long-term impact in terms of increased risk of various types of adverse social and health conditions, including poor social functioning, health and quality of life. Self-reported PTEs and torture in particular can serve as a ‘red flag’ to service providers of potentially diminished resilience, accumulated resource losses and increased difficulty in developing new resources. For torture survivors, erosion of interpersonal trust due to intentional harm seems to have resulted in a decreased confidence and trust in authorities and public service providers. This may in turn serve as an additional barrier to accessing services and limit disclosure without significant efforts in trust building. Very few participants in this study were using mental health services, despite an evident and in some cases expressed need. Torture survivors also seem to experience more discrimination and may be sensitized to further acts of felt injustice. Loneliness and social isolation were also more common in this group.

Early identification through systematic and effective screening should be the first step in guiding migrants and refugees suffering from experiences of torture and other PTEs to necessary services. The multiple needs of traumatized refugees and other migrants require flexible, multidisciplinary service provision. Services should emphasize continuous trust building through stable, long-term relationships with service providers while addressing linguistic, cultural and social barriers^14^ and strengthening resilience, personal strengths and social support.^38^

## Funding

This study was supported by the Finnish Cultural Foundation (AEC) and the Swedish Research Council for Health, Working Life and Welfare (Grant Number 2016-07194).

##  

*Conflicts of interest:* The authors declare that they have no conflicts of interest.

**Key points**

- Torture and other PTEs prevalent in refugee and migrant populations create a wide-ranging and long-term impact in terms of increased risk of various types of adverse social and health conditions.
- In this study, migrants that reported torture and other PTEs had significantly worse social and health outcomes.
- Torture survivors also reported lower trust and confidence in authorities and public service providers as well as more loneliness, social isolation and experiences of discrimination.
- Early identification through systematic and effective screening should be the first step in guiding migrants and refugees suffering from experiences of torture and other PTEs to flexible, multidisciplinary services.
